# Role of Various Extractants in Removing Group-IIB Elements of Soils Incubated with EDTA

**DOI:** 10.1100/tsw.2004.209

**Published:** 2004-12-06

**Authors:** Tanmoy Karak, Uttam Kumar Singh, D. K. Das

**Affiliations:** Department of Soil and Water Sciences, College of Resources and Environmental Sciences, China Agricultural University, Beijing-100094, P.R., China; ^2^ Department of Agricultural Chemistry and Soil Science, Faculty of Agriculture, Bidhan Chandra Krishi Viswavidyalaya, Mohanpur, Nadia-741252, West Bengal, India

**Keywords:** Gr-IIB elements (Zn, Cd, and Hg), extractants, EDTA (ethylenediaminetetraacetic acid), DTPA (diethylenetriaminepentaacetic acid), HCl, Mg(NO_3_)

## Abstract

This paper presents the results of an experimental investigation undertaken to evaluate different extractant solutions viz. HCl, Mg(NO_3_)^2^, and DTPA with the range of concentration from 0.001 to 0.1*N* after incubation with group-IIB metals (Zn, Cd, and Hg) and EDTA to understand the capability to remove Zn, Cd, and Hg from soils. Two noncontaminated soils, one acidic (GHL) and the other alkaline (KAP), in reaction were taken from an agricultural field of West Bengal, India for this investigation. Experiments were conducted on these two soils spiked with Zn^II^, Cd^II^, and Hg^II^ in concentrations of 612, 321, and 215 mg/kg for soil GHL and 778, 298, and 157 mg/kg for soil KAP, respectively, which simulate typical electroplating waste contamination. The removal of Zn, Cd, and Hg in soil GHL within the range of HCl concentrations was 8.2—16.5, 12.2—19.1, and 4.3—6.9 whereas these were 6.5—7.6, 8.5—14.1, and 3.2—5.2 in soil KAP. The removal of Zn, Cd, and Hg in soil GHL within the range of Mg(NO_3_)^2^ concentrations were 12.2—28.5, 19.1—24.6, and 18.2—19.1 whereas these were 9.1—12.1, 8.3—12.1, and 10.6—48.1 in soil KAP. For DTPA extractant, the percent removal of metal was found to be significantly higher than the other two extractants, which corroborates that DTPA is a better extractant for soil cleaning.

### INTRODUCTION

Zn, Cd, and Hg are present in group-IIB of the Periodic Table of which Zn is an essential micronutrient[1], but the other two have no beneficial effect for animals or plants. However, according to the USEPA[2], Zn is also a major public health concern at many contaminated sites. Although Zn is an essential micronutrient, all the elements present in group-IIB are referred to as heavy metals. Heavy metals are conventionally defined as elements with metallic properties (ductility, conductivity, stability as cations, ligand specificity, etc.) and an atomic number >20[3]. It is a remarkable contrast that, at one place, Zn is biologically one of the most important metals and is apparently necessary to all forms of life[4] and at another, Cd and Hg have no known biological role and are amongst the most toxic elements. All these metals are natural components of soil. Soil contamination resulting from improper waste disposal practices is a major concern.

Contamination, however, has resulted from industrial activities such as mining and smelting of metalliferous ores, electroplating, gas exhaust, energy and fuel production, fertilizer and pesticide application, and generation of municipal waste[5]. High levels of metals in soil can be phytotoxic. Poor plant growth and soil cover caused by metal toxicity can lead to metal mobilization in runoff water and subsequent deposition into nearby bodies of water.

Zn is readily adsorbed by clay minerals, carbonates, or hydrous oxides. Precipitation is not a major mechanism of retention of Zn in soils because of the relatively high solubility of Zn compounds. Cd may be adsorbed by clay minerals, carbonates, or hydrous oxides of Fe and Mn or may be precipitated as cadmium carbonate, hydroxide, and phosphate. Evidence suggests that adsorption mechanisms may be the primary source of Cd removal from soils[6]. Hg is introduced into the environment by both natural and anthropogenic processes, and, because of its volatility and solubility, is efficiently dispersed throughout the biosphere[7]. It is an environmental pollutant of concern not because of its direct impact on aquatic organisms, but because of their consumption by humans and other fish-eating mammals and birds.

Soil washing is the most commonly used treatment technology for the remediation of metal-contaminated soils[2]. This technique is an *ex situ* treatment method and is less expensive than many traditional or innovative treatment technologies, especially when smaller soil volumes are to be treated[2]. In this method, the contaminated soil is excavated and mixed with an extractant solution. The extractant solution may be water, acid, oxidizing agent, chelating agent, or surfactant, depending on the type of contaminant. The soil and the extractant solution are mixed thoroughly for a specified time, and the soil is dewatered to separate the soil and liquids. The resulting soil that meets regulatory requirements can be backfilled at the excavated site. The liquids that contain contaminants are further processed using conventional wastewater treatment technologies to recover the contaminants[2].

The environmental fate of chelating agents has received considerable attention. EDTA (ethylenediaminetetraacetic acid), for example, occurs at a higher concentration in river water than any other identified organic compound[8]. EDTA has been used as a chelating agent in the past to decontaminate nuclear reactors[9]. In agriculture, EDTA complexes have been used for about 30 years as commercial soil amendments to improve micronutrient availability. EDTA is a powerful hexadentate chelating ligand as metal carried in soils depends on their ability to keep these metals in soluble and mobile forms. To achieve this, the EDTA ligand must remain in solution and continue to complex with the metal ion present in soils. Attempts have been made to develop theoretical models from which the complexation of heavy metals by various chelates can be predicted and explained[10].

EDTA is persistent in the environment because it is resistant to decomposition by radiation[11] and rather slowly biodegradable in soil[12]. Bolton et al.[12] showed that only 15% of EDTA added to soil was degraded after 5 months, indicating that it could affect metals for a significant period of time. They also found that EDTA was not mineralized more rapidly or to a great extent in the surface soil than in the subsurface sediments. These results contrast with previous studies in which surface soils and organic C-enriched soils had greater rates and amounts of EDTA[13].

It is likely that chelate structure and/or the liability of the metal-chelate complex determine the resistance of the complex to mineralization. Diethylenetriaminepentaacetic acid (DTPA) and 0.1*N* HCl extraction procedures are commonly employed to estimate the plant-available forms of micronutrients in soil. They are used equally to determine available forms of heavy metals such as Cd and Hg[14]. 1*M* $Mg(NO_3)_2$ was chosen as an extractant because this solution extracts the most labile form of metals in soil without disturbing other fractions[15]. The presence of EDTA in soil may alter the mobility and transport of Zn, Cd, and Hg in soils because of the formation of water-soluble chelates, thus increasing the potential for metal pollution of natural waters. Mobility of metals is related to their extractability.

The aim of the experiment described here was to study the influence of EDTA on the extractability of Zn, Cd, and Hg by using HCl, $Mg(NO_3)_2$, and DTPA in both metal-amended soils. These studies provided valuable information on the distribution of Zn, Cd, and Hg in two soils of West Bengal, India and the removal of these metals using HCl, $Mg(NO_3)_2$, and DTPA after EDTA treatment. The present study was aimed to investigate the removal of Zn, Cd, and Hg from two diverse soils by using the above-mentioned extractant at different concentrations in order to determine an effective washing solution for the remediation of metal-contaminated soils.

### MATERIALS AND METHODS

#### Soils

Topsoils were taken from two different agricultural fields of West Bengal, India that were previously used for cultivation of rice. Soil samples were collected from the Ap horizon (0–20 cm). The samples were processed, passed through a 2-mm sieve, and analyzed for physical and chemical properties (Table 1) such as pH, organic C, clay, and $CaCO_3$ using standard analytical methods[16]. Cation exchange capacity (CEC) was calculated by the summation of exchangeable acidity and bases as proposed by Hendershot and Duquette[17]. Concentrations of plant-available Zn, Cd, and Hg in soil samples were determined using the DTPA method[18] and the total amounts of Zn, Cd, and Hg were estimated using the procedure described by Kuo et al.[19].

**TABLE 1**
**Physical and Chemical Properties of the Experimental Soils**

| Properties | | Soil | |
|---|---|---|---|
| | | Ghatal (GHL) | Kakdwip (KAP) |
| pH (1:2.5) | | 5.6 | 8.1 |
| Organic C, g/kg | | 5.2 | 1.6 |
| Clay, g/kg | | 284 | 66 |
| Textural class | | Silty clay loam | Silty loam |
| Taxonomic classification | | Typic Paleustalf | Typic Haplaquept |
| CEC [cmol($p^+$)/kg] | | 36.5 | 8.0 |
| $CaCO_3$ (%) | | 0.42 | 5.91 |
| DTPA, mg/kg | Zn | 0.56 | 0.08 |
| | Cd | ND† | ND |
| | Hg | ND | ND |
| Total metal, mg/kg | Zn | 1.25 | 2.53 |
| | Cd | 0.18 | 0.13 |
| | Hg | 0.08 | 0.02 |

†ND, not detectable.

#### Contaminants

The soils were spiked with Zn, Cd, and Hg to simulate typical electroplating waste constituents. The metals were applied in the form of $Zn(NO_3)_2.6H_2O$ for Zn, $Cd(NO_3)_2.4H_2O$ for Cd, and $Hg(NO_3)_2.H_2O$ for Hg on both soils. The soils were spiked by adding these chemicals dissolved in deionized water to the air-dried soils. The soils were equilibrated for 2 months and the resulting moisture content was 45% for these two experimental soils. After the preparation of the contaminant-spiked soils, they were mixed with $Na_2EDTA$ solution at the rate of 0.2 mg/kg soil to 300 g of soil samples. After being mixed well, each sample was split into three 100-g replications and put into plastic pots. The pots were placed in the greenhouse and the water content of the soil was adjusted to 70% of field capacity. In all treatments, pH was adjusted to soil-native pH by adding NaOH or $HNO_3$. Throughout the 6 months, water losses exceeding 10% of the initial values were compensated for by addition of distilled water.

### Soil Extractant and Extractant Procedure

Six months after the treatments, the soils were air dried, ground, and sieved through a 2-mm sieve. Total amounts of Zn, Cd, and Hg in simulated soils were estimated using $HNO_3$-$HClO_4$[19] (Table 2). The simulated soil samples were extracted for Zn, Cd, and Hg using different extractants viz. HCl, DTPA, and $Mg(NO_3)_2$. Three different concentrations (1*N*, 0.1*N*, and 0.01*N*) of HCl, $Mg(NO_3)_2$, and DTPA were used as extractant. Extraction was performed with a liquid to solids ratio (L/S) of 10:1. The mixture was stirred constantly for 2 h using a temperature-control orbital shaker. The 2-h mixing time was selected to investigate the easily removable Zn, Cd, and Hg fractions and to compare the relative removal efficiencies by various extractants. The soil solids were separated by centrifuging at 3000 rpm for 15 min and filtrated using a Whatman No.42 filter paper. The filtrate was analyzed for Zn, Cd, and Hg by atomic absorption spectrophotometry. Two of the three replicates were analyzed. If the data of two replicates were not within an acceptable range of precision (relative error <5% for high concentrations and <15% for low concentrations), the third sample was analyzed. This was necessary when the analyte was near the detection limit of the analytical procedure. Lastly, the removal of each contaminant was calculated using the following equation:

$$Contaminant \quad Removal\,(\%) = \frac{Contaminant\ Mass\ in\ Filtrate\,(C_L V_L)}{Initial\ Contaminant\ Mass\ in\ Soil\,(C_S M_S)} \times 100$$

where $C_L$ and $C_S$ are the concentrations of contaminant filtrate (mg/l) and soil (mg/kg), respectively. $V_L$ is the volume of supernatant (L) and $M_s$ is the dry mass of the soil (kg).

**TABLE 2**
**Total Amount of Zn, Cd, and Hg (mg/kg) Present in Contaminant-Spiked Soil**

| Soil | Zn | Cd | Hg |
|---|---|---|---|
| GHL | 612 | 321 | 215 |
| KAP | 778 | 298 | 157 |

## RESULTS AND DISCUSSION

### Extraction Using HCl

Table 3 shows the metal removals from both acidic (GHL) and alkaline (KAP) soils. In both soils, the percentage of metal removed increases with an increase of acid strength, however, these percentages of removal of metal were lower in alkaline soil. In GHL, Zn, Cd, and Hg removal ranged from 8.2–16.5, 12.2–19.1, and 4.3–6.9%, respectively. Whereas these were 6.5–7.6, 8.5–14.1, and 3.2–5.2% for Zn, Cd, and Hg in KAP soil, respectively. Thus, use of HCl decreases the removal percentage of group-IIB elements when one moves from top to bottom in the group except for Cd. This can be attributed to the significant covalent character of metal chloride, their comparatively low melting point, and by their layer-lattice (2D) crystal structures. In all cases, these may be regarded as close-packed lattices of chloride ions in which the $Zn^{II}$ ions occupy tetrahedral, $Cd^{II}$ ion octahedral, and $Hg^{II}$ ion elongated octahedral site. The structure of $CdCl_2$ is also important since it is typical of $MX_2$ (where X = F, Cl, Br, or I) compound and its marked polarization effects are expected[20]. In both the soils, the removal percentage of group-IIB metal follows Cd > Zn > Hg. The lower percentage of Hg removal can be explained by the fact that the covalency is still more pronounced in $HgCl_2$ than in the corresponding chlorides of Zn and Cd. The crystalline structure of $HgCl_2$ is also an important factor for its solubility in any solvent medium[21]. The crystalline structure of $HgCl_2$ is comprised of linear Cl—Hg….Cl molecules in which radii of Cl—Hg is 225 pm and the next shortest Hg….Cl distance is 334 pm[21]. Therefore, structure and covalence of group-IIB metal chloride is an important factor in their removal by HCl extractant.

**TABLE 3**
**Removal of Zn, Cd, and Hg (%) from Contaminated-Spiked Soils Using HCl**

| Soil | Contaminant | Strength of HCl | | |
|---|---|---|---|---|
| | | 0.001*N* | 0.01*N* | 1*N* |
| GHL | Zn | 8.2 | 10.1 | 16.5 |
| | Cd | 12.2 | 18.9 | 19.1 |
| | Hg | 4.3 | 5.2 | 6.9 |
| KAP | Zn | 6.5 | 6.9 | 7.6 |
| | Cd | 8.5 | 12.3 | 14.1 |
| | Hg | 3.2 | 4.8 | 5.2 |

### Extraction Using $Mg(NO_3)_2$

Table 4 summarizes the metal removal efficiencies of $Mg(NO_3)_2$ from the soils at different concentrations. For soil GHL, the percentages of Zn and Cd removed was found maximum when 0.01*N* $Mg(NO_3)_2$ was applied as an extractant and they were 28.5 and 24.6%, respectively. In the case of Hg, the amount of Hg removed increases with an increase of extractant strength in both soils. However, the percentage of Hg removed from soil KAP was found to be significantly higher than GHL. For soil KAP, the percentages of removed Zn and Cd decrease with an increase of extractant strength. It should be noted that extractability of $Mg(NO_3)_2$ is higher than HCl. Table 4 shows that $Mg(NO_3)_2$ has a strong capability to extract Hg in alkaline soil. The nitrate group ($-NO_3$) is a versatile ligand and numerous modes of coordination have been found in nitrato complexes[22]. When more sites become available, or when the coligands are less bulky, then the nitrate group become bidentate bridging, which forms a more extensive network of bridging nitrate group[20]. This might be the reason for the removal of a high percentage of Zn, Cd, and Hg at 0.01*N* $Mg(NO_3)_2$ in soil GHL.

**TABLE 4**
**Removal of Zn, Cd, and Hg (%) from Contaminated-Spiked Soils Using $Mg(NO_3)_2$**

| Soil | Contaminant | Strength of $Mg(NO_3)_2$ | | |
|---|---|---|---|---|
| | | 0.001*N* | 0.01*N* | 1*N* |
| GHL | Zn | 14.1 | 28.5 | 12.2 |
| | Cd | 19.1 | 24.6 | 23.1 |
| | Hg | 18.2 | 18.9 | 19.1 |
| KAP | Zn | 12.1 | 9.8 | 9.1 |
| | Cd | 12.1 | 8.5 | 8.3 |
| | Hg | 10.6 | 18.9 | 48.1 |

**TABLE 5**
**Removal of Zn, Cd, and Hg (%) from Contaminated-Spiked Soils Using DTPA**

| Soil | Contaminant | Strength of DTPA | | |
|---|---|---|---|---|
| | | 0.001*N* | 0.01*N* | 1*N* |
| GHL | Zn | 35 | 49 | 64 |
| | Cd | 32 | 47 | 62 |
| | Hg | 48 | 72 | 81 |
| KAP | Zn | 39 | 59 | 71 |
| | Cd | 39 | 52 | 67 |
| | Hg | 51 | 78 | 89 |

### Extraction Using DTPA

Table 5 shows the metal removal from both soils using DTPA at different concentrations. Chelating agents modify metal concentrations in soil solution by forming various soluble complexes, thus enhancing metal removal[23]. In both soils, the percentage of metal removal increases with an increase of DTPA concentrations, however, the percentage of metal removed from KAP soil was higher than GHL soil. The percentages Zn, Cd, and Hg in soil GHL ranged from 35–64, 32–62, and 48–81 whereas these were 39–71, 39–67, and 51– 89 in soil KAP. The Zn, Cd, and Hg removal increases significantly using DTPA when compared with the use of HCl or $Mg(NO_3)_2$ as extractants. This may be due to the fact that these metals form more stable complexes with DTPA and more solubilized M-DTPA. The soils were contaminated with Zn, Cd, and Hg followed by incubation with EDTA. The log stability constants of M-EDTA and DTPA complexes (M = Zn, Cd, and Hg) are shown in Table 6. From Table 6 it is confirmed that the stability of EDTA and DTPA complexes follows the sequence Hg > Zn > Cd. After addition of DTPA, M-EDTA complexes shift to M-DTPA complexes as M-DTPA complexes are more stable than M-EDTA. As the conditions become progressively more alkaline in soil KAP, $H^+$ ions begin to dissociate from the DTPA molecule’s five carboxyl groups, eventually leaving seven pairs of electrons (five pairs from the carboxyl groups and two nitrogen pairs) that are available for bonding with a metal cation. At high pH, soil KAP bears negative charge and metal-DTPA complex also bears the same charge, which increases the possibilities of the migration of metal-DTPA complexes from soil-to-soil solution. Another reason to favor M-DTPA complex formation is that DTPA is a stronger chelating agent than that of

**TABLE 6**
**Stability Constants ($\log K^{c}_{0.1}$) at 25ºC for Group-IIB Metal-EDTA and DTPA Reactions[24]**

| Reaction | Ligand (L) | |
|---|---|---|
| | EDTA | DTPA |
| Zn+L $\leftrightarrow$ ZnL | 16.44 | 18.29 |
| Zn+H+L $\leftrightarrow$ ZnHL | 19.44 | 23.89 |
| Cd+L $\leftrightarrow$ CdL | 16.36 | 19.00 |
| Cd +H+L $\leftrightarrow$ CdHL | 19.26 | 23.17 |
| Hg+L $\leftrightarrow$ HgL | 21.50 | 26.40 |
| Hg +H+L $\leftrightarrow$ HgHL | 24.60 | 30.68 |

EDTA. DTPA forms complexes with metal ions most efficiently in basic solutions[25]. The degree of complexation depends on the pH of the aqueous system. At low pH values, the DTPA exists in the acid form or positive charge species and does not chelate effectively because hydrogen ions occupy the coordinating functional groups. As the pH is increased, the DTPA reaches a maximum chelating ability as DTPA becomes fully deprotonated because of negative charge generation as carboxylate ion, $-COO^-$, from carboxylic acid group, which corroborates that the removal of metal from KAP is always in higher than GHL by DTPA. The covalent radii of Zn, Cd, and Hg are 131, 148, and 149 pm, respectively[26]. Therefore, soft character increases from Zn to Hg. According to the hard/soft acid base principle, hard acids prefer to associate with hard bases, and soft acids prefer soft bases[27]. The almost invariable oxidation state of Zn and Cd is +2 and in aqueous solution they are appreciably hydrolyzed to species such as $[M(OH)(H_2O)_x]^+$ and $[M_2(OH)(H_2O)_x]^{3+}$ and a number of basic salts such as $ZnCO_3.2Zn(OH)_2.H_2O$ and $CdCl_2.4Cd(OH)_2$ can be precipitated. But in contrast, $Zn^{II}$ and $Cd^{II}$ compounds hydrolyze rapidly in water, no doubt because of the ability of $Zn^{II}$ and $Cd^{II}$ to increase their coordination number above 4 and form DTPA complexes. However, in the case of Hg, the ability to form DTPA complex is much more pronounced because of its characteristic ability to form not only conventional ammine and amine complexes, but also the predominant chelate effect[20]. Overall, these results show that the formation of soluble M-DTPA complexes in KAP at soil-native pH may provide improved removal efficiency of metal in the presence of multiple metals, and the effects of soil pH at high concentrations of DTPA on metal removal require further investigation.

The results of the Pearson correlation (Table 7) of three extractants showed that DTPA-HCl and DTPA-$Mg(NO_3)_2$ show the significant correlation in both soils for all metal removal. However, there were no significant correlations between HCl and $Mg(NO_3)_2$ extractants for Zn and Hg in soils GHL.

**TABLE 7**
**Pearson Correlation Coefficients Between Extractant Methods for Metals**

| | GHL | | | KAP | | |
|---|---|---|---|---|---|---|
| | **Zn** | **Cd** | **Hg** | **Zn** | **Cd** | **Hg** |
| DTPA-HCl | 0.981* | 0.996* | 0.989 | 0.981* | 0.919* | 0.966* |
| DTPA-$Mg(NO_3)_2$ | 0.991* | 0.892* | 0.899* | 0.956* | 0.798* | 0.874* |
| HCl-$Mg(NO_3)_2$ | 0.216 $^{NS}$ | 0.699* | 0.213 $^{NS}$ | 0.869* | 0.925* | 0.925* |

*Significant at the 0.05 probability levels; $^{NS}$ Not significant at the 0.05 probability level.

### CONCLUSIONS

This experiment exhibits that the overall removal of Zn, Cd, and Hg was higher when DTPA was used as the extractant as compared to the other extractants, HCl and $Mg(NO_3)_2$. From this experiment, it can be said that removal of Zn, Cd, and Hg from the soils should be an important consideration in designing a soil-washing system. HCl and $Mg(NO_3)_2$ were not as effective in removing Zn, Cd, and Hg as DTPA, but $Mg(NO_3)_2$ was found to be more effective than HCl to remove these metals from soils. Besides, this study showed that complete removal of Zn, Cd, and Hg from these two soils is difficult when trying to achieve a complete soil-washing process. Additionally, the use of one extractant may not be effective in removing all the metals from the soils, therefore, a sequence of extractants may be needed for the removal of multiple metals of group-IIB contaminants from soils.
